# Antifatigue Functions and Mechanisms of Edible and Medicinal Mushrooms

**DOI:** 10.1155/2017/9648496

**Published:** 2017-08-14

**Authors:** Ping Geng, Ka-Chai Siu, Zhaomei Wang, Jian-Yong Wu

**Affiliations:** ^1^Department of Applied Biology & Chemical Technology, State Key Laboratory of Chinese Medicine and Molecular Pharmacology in Shenzhen, The Hong Kong Polytechnic University, Hung Hom, Kowloon, Hong Kong; ^2^School of Food Science & Engineering, South China University of Technology, Guangzhou 510640, China

## Abstract

Fatigue is the symptom of tiredness caused by physical and/or psychological stresses. As fatigue is becoming a serious problem in the modern society affecting human health, work efficiency, and quality of life, effective antifatigue remedies other than pharmacological drugs or therapies are highly needed. Mushrooms have been widely used as health foods, because of their various bioactive constituents such as polysaccharides, proteins, vitamins, minerals, and dietary fiber. This paper reviews the major findings from previous studies on the antifatigue effects, the active components of mushrooms, and the possible mechanisms. Many studies have demonstrated the antifatigue effects of edible and medicinal mushrooms. These mushrooms probably mitigate human fatigue through effects on the functional systems, including the muscular, cardiovascular, hormone, and immune system. The bioactive constituents that contribute to the antifatigue effects of mushrooms may include polysaccharides, peptides, nucleosides, phenolic compounds, and triterpenoids. Further research is still needed to identify the active ingredients and to investigate their mechanism of action on the antifatigue effects. Since most previous studies have been carried out in animal models, more human trials should be performed to verify the antifatigue function of edible and medicinal mushrooms.

## 1. Introduction

Fatigue is the physical or mental exhaustion caused by overwork, exercise, and lack of sleep. It can also be a symptom resulting from medicine, illness, anxiety, or depression. Fatigue affects more than 20% of people worldwide, which is usually associated with physical and/or psychological (mental) weakness [1]. For physical weakness, the individuals engaged in vigorous activities or laborious jobs may experience reduced efficiency and capacity for work. For psychological weakness, people with depression or sleep disorders may have the sense of weariness, exhaustion, and lack of motivation [2]. As fatigue can be misunderstood as laziness or lethargy, some people may not recognize it as a serious problem to seek proper treatment. The stimulants such as coffee, energy drinks, and even caffeine or ephedrine supplements taken by many people can only provide a temporary relief and even create health problems in the long run. Therefore, it is imperative to resolve fatigue through fundamental and constructive measures for repairing the dysfunction of the responsible body systems [3].

There is a long history for the medicinal use of mushrooms or their active components. Many research studies have demonstrated the various health benefits of edible and medicinal mushrooms such as antioxidant, anticancer, prebiotic, immunomodulating, anti-inflammatory, cardiovascular, antimicrobial, and antidiabetic activities [4–8]. Some mushrooms have also been shown to provide antifatigue functions by balancing various biological systems and helping to maintain the basic harmonious pattern of body. Meanwhile, previous studies have identified the major active constituents responsible for the biological effects of edible and medicinal mushrooms with polysaccharides being the most common and abundant [9, 10]. Other bioactive compounds found in the mushrooms include peptides [11] and various organic molecules such as triterpenoids [12], nucleosides [13], phenolics, and flavonoids [14].

This review aims to summarize the recent studies on the antifatigue effect of edible and medicinal mushrooms. The physiological basis of fatigue is introduced with respect to the exhaustion theory, the radical theory, the clogging theory, and the hemoglobin theory. The antifatigue mechanism of edible and medicinal mushrooms is illustrated from several aspects including muscular function, antioxidant effect, cardiovascular function, immunomodulation, hormone regulation, hepatic function, and blood glucose regulation. The bioactive constituents responsible for the antifatigue functions are summarized.

## 2. Physiological Basis of Fatigue

Fatigue in medical term can be classified into chronic fatigue syndrome (CFS), central fatigue, and peripheral fatigue. CFS is a kind of neurological illness, which may be caused by abnormal function of immune system and neuroendocrine system, infections, or nutritional deficiency [15]. Patients suffering from CFS are in a serious medical condition and not easily recovered by rest. Central fatigue is associated with changes in the synaptic concentration of neurotransmitters, primarily serotonin, within the central nervous system (including the brain and spinal cord) [16]. For healthy individuals, central fatigue can occur after prolonged exercise and exerts a protective effect reducing muscle contraction to lower oxygen tension in brain and helps to balance various organs for normal functioning [17]. It is not directly linked with the level of physical exercise and thus cannot be explained by factors affecting muscle function. Peripheral fatigue mainly refers to physical fatigue, corresponding to “an inability to continue exercise at the same intensity with a resultant deterioration in performance” [18]. There are several theories explaining the physiological basis of peripheral fatigue, including the exhaustion theory, the radical theory, the clogging theory, and the hemoglobin theory.

Exhaustion theory suggests that fatigue results from exhaustion of liver and muscle glycogen reserves and significant decrease of blood glucose concentration [19]. Glucose is the primary source of energy for body cells, which is oxidized to generate ATP during cell respiration. The glucose not used as fuel will immediately travel to and be stored in liver or skeletal muscles in the form of glycogen or stored as fat in the adipose tissue. The stored glycogen in liver can convert back to glucose which will be released into bloodstream when blood glucose level drops. Under prolonged work or exercise, the glucose derived from stored muscle and liver glycogen will be insufficient, requiring additional energy produced from the oxidation of fat. However, the process of fat oxidation is difficult to supply high level of energy. People will experience fatigue when the glycogen stored in muscles and liver is depleted [20].

Radical theory indicates that free radicals such as hydroxyl and superoxide anion radicals will accumulate in the body after intense physical load and put the body in a state of oxidative stress because of imbalance between the body's oxidation system and antioxidation system. These free radicals, referred to as reactive oxygen species (ROS), will disturb the normal redox state in the cells, resulting in damage of cell components including lipids, proteins, and DNA, dysfunction of cellular organs, and poor energy metabolism [21, 22].

Clogging theory illustrates that the accumulation of lactic acid and intracellular inorganic phosphate (Pi) resulting from anaerobic respiration will affect cellular homeostasis and result in fatigue. When the maximal aerobic capacity is reached, the oxygen supply for oxidative phosphorylation of ATP production is insufficient. As a result, anaerobic respiration with nonoxidative phosphorylation increases to meet the body need, leading to the accumulation of lactic acid intracellularly. The H^+^ ions from lactic acid inhibit energy metabolism as the key enzymes involved in glycolysis and gluconeogenesis are inhibited under low pH [23]. Apart from this, the acidosis will inhibit calcium ion pumping in sarcoplasmic reticulum and consequently reduce the degree of muscle contractile activation. Also, when burst of energy is needed for muscle, the hydrolysis of creatine phosphate which gives creatine and Pi will be involved in the anaerobic respiration. The muscle contraction force will decrease as Pi level increases, leading to the feeling of fatigue [24].

Hemoglobin theory suggests that cells and tissues may be damaged during strenuous exercise due to destruction of red blood cells caused by leakage of energy metabolic system coenzyme and myoglobin [25]. The destructed red blood cells cannot transport oxygen to the muscular tissues successfully, disturbing the production of ATP by aerobic respiration and causing fatigue.

## 3. Mechanisms for Antifatigue Effects of Edible and Medicinal Mushrooms

### 3.1. Muscular Function

The level of fatigue could be evaluated based on the energy metabolism of muscles [26]. Exercise endurance time is an important and straightforward variable in evaluating the antifatigue effect of edible and medicinal mushrooms. Swimming is the most commonly accepted experimental exercise model to evaluate the extent of muscular fatigue [27]. The swimming endurance time is normally defined as the time when mice keep the swimming activity until they sink into the bottom of swimming pool and stop moving for a certain time period (e.g., 10 s) [28]. Other exercise models including rotating rod test and forced running test have also been adopted to evaluate the level of fatigue [29]. Edible and medicinal mushrooms function to mitigate muscular fatigue by affecting blood lactic acid and glycogen storage, blood urea nitrogen level, relevant enzyme activities, metabolic regulators, and expression of relevant transporters.

#### 3.1.1. Lactic Acid and Glycogen Storage

Lactic acid is the product of glycolysis under anaerobic condition. The accumulation of lactic acid during exercise will inhibit energy metabolism and reduce muscular endurance, resulting in fatigue. Glycogen storage in liver and muscle is an important energy source to supply sufficient fuel for exercise. The hepatic glycogen can be converted into glucose and released into blood for energy supply when blood glucose level is low [30]. Therefore, blood lactic acid, muscle, and liver glycogens are all important indicators judging the degree of fatigue [28, 31]. Edible and medicinal mushrooms have been demonstrated to mitigate fatigue through inhibiting blood lactic acid generation and increasing glycogen storage in liver and muscle. For instance, T. Li and W. Li found that the water extracts of* Cordyceps sinensis* can inhibit the production of lactic acid during exercise and increase liver and muscle glycogen storage with the dosage of 200 mg/kg polysaccharides from* C. sinensis* most effective in mice [32]. The extracts of submerged fermentation of* Ganoderma lucidum* were also found to inhibit the accumulation of blood lactic acid, accelerate lactic acid clearance, improve glycogen reserve, and reduce glycogen consumption during exercise, resulting in less fatigue [33].

#### 3.1.2. Blood Urea Nitrogen

The blood urea nitrogen (BUN) is the nitrogen content in urine from the breakdown of proteins, which occurs when energy production from sugar and fat metabolism is insufficient to support the body. The body will remove such waste product from protein breakdown in order to maintain normal functioning of the body system. Therefore, the BUN level is also an important indicator showing the capability of body to suffer from physical load and fight against fatigue [34]. Many scholars use the BUN level to exhibit the antifatigue effect of edible and medicinal mushrooms. For instance,* G. lucidum* (directly used as powder with 60 g/kg feed) can reduce the decomposition of protein for energy with BUN level decreased from 42.0 mg/dl of control to around 31.5 mg/dl after two-week treatment, indicating that* G. lucidum* can enhance energy metabolism from carbohydrate and fat to supply enough energy in order to limit protein breakdown [35]. Wu et al. found that the* Hirsutella sinensis* can also extend swimming time to exhaustion in rats and reduce blood lactate and BUN levels but did not have an effect on tissue glycogen [36]. Wu and coworkers found that the mycelium hot water extracts of* Ophiocordyceps sinensis* could increase the swimming endurance of mice up to 100% and also increase glycogen level and reduce lactic acid and BUN levels significantly [37]. The water extracts of* Agaricus bisporus* with the dosage of 120 mg/kd·d in mice also showed effect on BUN reduction of about 46% compared with the control group, indicating better nutrient circulation to avoid fatigue [38].

#### 3.1.3. Lactate Dehydrogenase Activity

Lactate dehydrogenase (LDH) is an enzyme found in almost all body tissues involved in cellular respiration. It is involved in the process known as the Cori cycle. The increase in LDH activity can help to produce enough ATP for exercise under anaerobic condition and speed up the removal of lactic acid [35]. Liang and coworkers found that the LDH activity can be significantly stimulated by* G. lucidum*. Compared with the control group with LDH activity of around 442 U/dl, the* G. lucidum* treated mice group had LDH of around 518 U/dl. The LDH activities of other edible and medicinal mushrooms were also compared by Liang et al. together with the BUN level, lactic acid level, and swimming time (Table 1). Results show that* G. lucidum*,* C. sinensis*,* Lentinus edodes*,* Tremella fuciformis*, and* Hericium erinaceus* could all effectively increase swimming endurance and reduce BUN and lactic acid level, with* G. lucidum* and* C. sinensis* showing most significant stimulating effect on LDH activity [35].

#### 3.1.4. Skeletal Muscle Metabolic Regulators and Transporters

**Figure 1:**
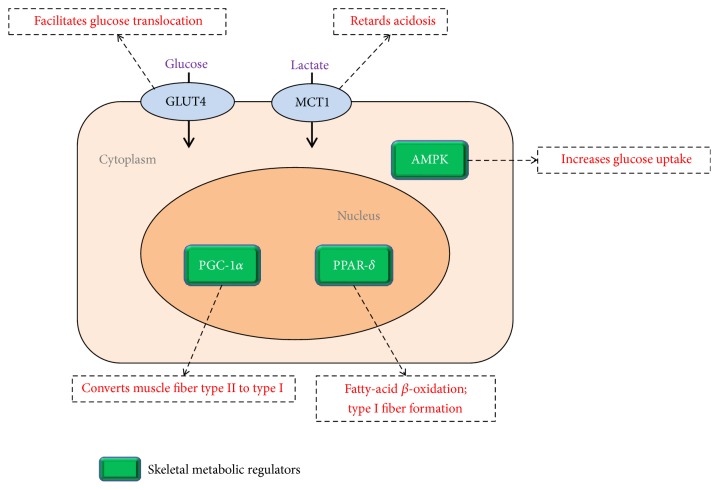
A possible mechanism with skeletal muscle metabolic regulators and transporters for the muscle endurance enhancement with a medicinal mushroom* C. sinensis* (adapted from [39]).

Kumar and coworkers studied the antifatigue activity of* C. sinensis* in the molecular level and found that* C. sinensis* could enhance muscle endurance by promoting the expression of skeletal muscle key metabolic regulators AMPK, PGC-1*α*, and PPAR-*δ* (Figure 1) [39]. These genes could induce marked changes in skeletal muscle metabolism, including increases in glycogen breakdown, glycolysis, glucose uptake, and oxidation of fatty acid, together with many changes in gene expression promoting endurance [40]. AMPK is an AMP-activated protein kinase, which was activated in muscle during exercise and electrically stimulated contraction. High AMPK activity can lead to higher basal glucose uptake [40]. PGC-1*α* is a transcriptional coactivator that can activate the oxidative metabolism and biogenesis in mitochondria. The activation of PGC-1*α* drives the conversion of muscle fiber type II to type I fibers which are slow twitch muscle fibers with higher oxidative capacity. They can use oxygen more efficiently to produce more ATP for continuous muscle contractions and are more resistant to fatigue [41]. PPAR-*δ* is the transcriptional regulator for fatty acid oxidation in adipose tissue by activating the enzyme associated with long chain fatty acid *β* oxidation. It is able to drive fatty acid transport and oxidation, increase the formation of type I muscle, and enhance heart contraction function and thus can improve physical endurance and performance [42].

Meanwhile, Kumar et al. [39] also found that* C. sinensis* was capable of stimulating the expression of lactate monocarboxylate transporter MCT1 and glucose transporter GLUT4 (Figure 1). When lactic acid accumulation occurs during exercise, it dissociates to form lactate and hydrogen ions, leading to acidosis and fatigue. Lactate can help to retard acidosis and inhibit fatigue because it acts as a buffer to the protons accumulated during exercise. MCT1 can facilitate the lactate transport into skeletal muscle. GLUT4 is a glucose transporter found in adipose tissue and striated muscle. It is induced by insulin signal to facilitate the translocation of glucose into cell. Therefore, the effective expression of MCT1 and GLUT4 could retard acidosis and enhance glucose transport in skeletal muscle, so that muscle endurance could be improved [39]. The medicinal mushroom of* Phellinus linteus* has also been found to increase endurance exercise performance through increasing the expression of MCT1 and MCT4 in muscles [43].

### 3.2. Antioxidant Effect

#### 3.2.1. Free Radical Scavenging Activity

Reactive oxygen species (ROS) is the byproduct from the oxidative reaction of ATP production in mitochondria. Although organisms can generate antioxidant and have repair systems that can protect them from oxidative damage, the condition of fatigue will weaken the antioxidant system, resulting in excess free radicals and increase of oxidative stress. The oxidative stress of cells reduced by the free radical scavenging ability of edible and medicinal mushrooms can help to enhance energy metabolism in mitochondria, which can accelerate energy production rate and help to mitigate fatigue. The antioxidant activity of edible and medicinal mushrooms could be evaluated* in vitro* through the scavenging of 2,2-diphenyl-1-picrylhydrazyl (DPPH), hydroxyl radical, superoxide anion radical, nitric oxide, and the inhibition effect on lipid peroxidation. The hydroxyl and superoxide anion could be generated by the mitochondria during respiration, which may impair cellular structures and functions if they are produced in excess. Nitric oxide could react with the superoxide anion generated and form peroxynitrite. Peroxynitrite is a highly reactive oxidant and nitrating agent, which could damage a wide array of cellular molecules including proteins and DNA. Lipid peroxidation is the oxidative degradation of lipids in cell membranes, which also causes cell damage [45].

The antioxidant activities of edible and medicinal mushrooms are evaluated based on the above-mentioned aspects. For instance, researchers found that the hot water extracts of* C. sinensis* exhibited DPPH, hydroxyl, and superoxide anion radical scavenging activity with the inhibition effects increasing with doses [46]. Joseph and coworkers found that the chloroform extracts of* G. lucidum* were able to scavenge the reactive oxygen species and enhance body antioxidant defense. With 1000 *μ*g/ml chloroform extract of* G. lucidum*, the DPPH scavenging activity could reach around 91.1%. Meanwhile, the chloroform extract could also inhibit lipid peroxidation and scavenge superoxide anion and nitric oxide, preventing the cell membrane and cellular molecules from damage [47]. The studies of Chen suggested that polysaccharides from* T. fuciformis* also had scavenging activity on the superoxide anion and hydroxyl radical, which can effectively reduce the formation of highly reactive oxidant peroxynitrite and mitigate biomolecules damage. The superoxide scavenging activity increased from 17 to 90% with increasing polysaccharide concentration in* T. fuciformis* from 0.1 to 0.4 mg/ml [48].

#### 3.2.2. Enhancing Antioxidant Enzyme Activity

Researchers found that edible and medicinal mushrooms can stimulate the activities of antioxidant enzymes, such as superoxide dismutase (SOD), glutathione peroxidase (GSH-Px), glutathione reductase, and catalase, to prevent oxidative stress and fatigue. SOD is a natural enzyme that can neutralize specifically superoxide radicals by utilizing different positively charged metal ions like copper or zinc. GSH-Px is also a natural antioxidant enzyme which can scavenge and inactivate hydroxyl radicals. Glutathione reductase and GSH-Px make up the glutathione system of antioxidant enzymes to reduce lipid hydroperoxides and free hydrogen peroxides. Catalase is an antioxidant enzyme working with SOD to prevent free radical damage. SOD converts superoxide radicals to hydrogen peroxides and catalase converts them into harmless water and oxygen [49].

Ma et al. stated that polysaccharides from* T. fuciformis* can stimulate the formation and activity of the enzymes of SOD and GSH-Px, demonstrating potent antioxidant activity of* T. fuciformis* to prevent oxidative stress [50]. The polysaccharides from* L. edodes* were figured out to stimulate the activity of SOD and GSH-Px. Besides, the blood malondialdehyde (MDA) level of* L. edodes* treated mice was lower than that of the control group, indicating that the oxidative damage resulting from ROS attack was limited. Therefore,* L. edodes* can help to limit oxidative damage on cell effectively [49]. The water extracts of* A. bisporus* were also found to significantly stimulate the activities of catalase, glutathione reductase, and GSH-Px, which contributed to the antioxidant effect of* A. bisporus* [51].

Through effective scavenging of free radicals and enhancement of antioxidant enzyme activities, edible and medicinal mushrooms could protect tissues from exercise-induced oxidative damage, thus reducing physical fatigue.

### 3.3. Cardiovascular Function

The cardiovascular system includes the heart and two networks of blood vessels. The contraction of heart provides an arterial blood pressure to push blood throughout the body. The circulation of blood through blood vessels network transports nutrients and oxygen to individual cells and removes waste products for disposal [52]. The proper functioning of cardiovascular system is one of the essential factors to prevent fatigue.

Edible and medicinal mushrooms can promote and enhance blood circulation by vasodilation, that is, the expansion of blood vessels when the smooth muscles of vessel wall relax. The dilation of blood vessel can be induced by vasodilators in single organ or throughout the entire body. Therefore, vasodilation can be controlled to occur in areas that need blood supply increase, helping the body to keep tissues alive and function properly [52].

*C. sinensis* is a typical medicinal mushroom which has been found to have vasodilation effect on blood vessels. It helps to avoid fatigue by redistribution and increase blood flow to supply enough oxygen and nutrients to essential organs and muscles. Feng and coworkers found that the aqueous phosphate buffer saline extracts of* C. sinensis* can stimulate nitric oxide production and endothelium-derived hyperpolarizing factor (EDHF) to reduce arterial pressure and induce the endothelium-dependent vasodilation effect [53]. The active component of adenosine in* G. lucidum* has also been found as an important contributor to vasodilation, which can reduce blood viscosity, inhibit platelet aggregation, and improve blood oxygen supply capacity [54]. When body is supplied with sufficient energy from the cardiovascular system, the condition of fatigue could usually be effectively alleviated.

### 3.4. Immunomodulation

The immune system is a complex system that can remember and recognize pathogens and produce cells and secretions to remove them with high specificity. The active substances in edible and medicinal mushrooms can improve the immune system by activating immune effector cells including macrophages, natural killer cells, and T cells. A well-performed immune system can ensure proper functioning of other body systems and adequate amount of energy supply to each part of body so as to prevent fatigue. The immunomodulation effect of edible and medicinal mushrooms is achieved mainly through the following mechanisms.

#### 3.4.1. Activation of Macrophages

Macrophages are white blood cells constituting the first-line defense of body to engulf foreign invaders. In the studies of Wang et al.,* G. lucidum* can activate macrophages, lymphocytes, and other immune cells to respond to pathogens [55]. Jia and Lau also found that* C. sinensis* could enhance phagocytosis of macrophages, which removed foreign pathogens and triggered specific activities of other immune cells more effectively [56].

#### 3.4.2. Increase of T Helper Cells and Ratio of T Helper: T Suppressor Cells

T helper cells are one kind of lymphocytes that have no cytotoxic or phagocytic activity to kill pathogens or infected cells. They play an important role in the immune system which controls and regulates the other immune cells to respond to foreign invaders. The T suppressor cells are a subpopulation of T cells that can maintain homeostasis of the immune system and avoid immune response towards self-antigen. The increase in T helper: T suppressor cells means enhancement in immune system activity. It has been found by Chen and coworkers that the number of T helper cells and ratio of T helper: T suppressor cells in mice can be increased by treatment with the ethanol extracts of* C. sinensis* [57].

#### 3.4.3. Increase of Natural Killer Cells Activity and Cytokines Expression

Natural killer cells belong to cytotoxic lymphocytes which have their own specific mechanism to identify foreign invaders and remove them. They can kill infected cells or even cancer cells by triggering various cell destroying mechanisms. Cytokines are a category of signaling molecules used for cellular communication. They are released by different immune cells to regulate immune response in infective sites.* C. sinensis* has been proved to enhance the activity of natural killer cells and increase the production of cytokines [57]. Mao and coworkers figured out that* G. lucidum* can stimulate the expression of cytokines and the proliferation of T lymphocytes [58]. Wu et al. found that dietary supplementation with* A. bisporus* can enhance the activity of natural killer cells and the production of cytokines as well [59].

The immunomodulation effect of edible and medicinal mushrooms helps to ensure a well-performed immune system for the whole body to function properly so as to lower the occurrence of fatigue.

### 3.5. Hormone Regulation

Hormone is a chemical released by cells or glands, which is responsible for message transfer to affect cells in other parts of the body. Testosterone is a steroid hormone primarily secreted by the Leydig cells of men testes and women ovaries. High testosterone level leads to improvement in muscle development and high energy level. However, significantly high testosterone level also causes side effects including dysfunction of liver and cardiovascular system [60]. The active compounds in edible and medicinal mushrooms can either simulate or inhibit testosterone production depending on the mushroom species, which helps to balance the testosterone level and maintain body energy level to prevent fatigue.

Different medicinal mushrooms have different effect on the production of testosterone. Huang and coworkers suggested that* C. sinensis* can stimulate the production of testosterone in mice Leydig cells through the stimulation of steroidogenesis with 3 mg/ml inducing the maximal production rate [61].* G. lucidum* was found to exhibit hormone regulation activity, which reduces testosterone activity when it is abnormally high and affects normal energy metabolism [62].

### 3.6. Hepatic Function

Liver is a very important organ responsible for many critical functions. Glucose is stored in liver in the form of glycogen. The liver will release glucose when body needs extra amount of energy. The liver also acts as blood filter to remove toxins in blood and produce cleaner blood which can carry more oxygen and nutrients. The fat metabolism of liver is also important to maintain healthy cardiovascular system and body weight. All these functions of liver are closely related to the proper functioning of body. The dysfunction of liver will have a negative impact on energy metabolism and can therefore lead to fatigue. Edible and medicinal mushrooms have been reported to improve hepatic function from the following aspects.

#### 3.6.1. Increase of Liver Energy State

The study of Manabe et al. found that the hot water extracts of* C. sinensis* can increase liver ATP to Pi ratio which represents high energy state of liver, and increase liver blood flow. The* C. sinensis* extracts can increase the energy content in liver cell because they induce the relaxation of liver blood vessels, leading to increase in liver blood flow. In this case, the liver can work effectively to promote blood circulation and clean blood to carry more oxygen and nutrient for energy metabolism in mitochondria [63].

#### 3.6.2. Promotion of Liver Protein and RNA Synthesis

Edible and medicinal mushrooms have also been found to promote protein and RNA synthesis, which helps to maintain the normal function of liver and avoid fatigue. For instance, Y. Wei and C. Zheng reported that* T. fuciformis* can promote the leucine incorporation into liver protein and orotic acid incorporation into RNA in liver cells of mice [64].

### 3.7. Blood Glucose Regulation

The blood glucose level is maintained through the actions of two pancreatic hormones, insulin and glucagon. Insulin functions to reduce blood glucose back to normal by stimulating body cells to increase glucose uptake rate, increase cell glucose metabolism, increase glycogen formation from glucose in liver, and increase fat synthesis from glucose in liver and adipose cells. Glucagon works in the opposite way to increase blood glucose to the normal level. Abnormally high blood glucose level may lead to hyperglycemia and diabetes which can result in heart disease or kidney failure, while abnormally low blood glucose level may lead to hypoglycemia which is usually associated with chronic fatigue.

**Figure 2:**
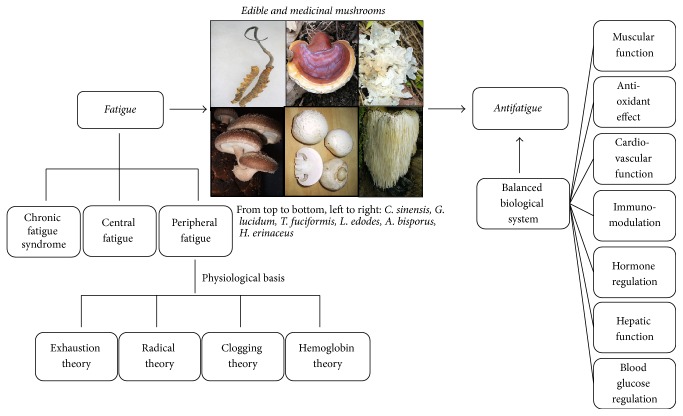
Illustration of physiological basis of fatigue and antifatigue mechanisms of edible and medicinal mushrooms (photos obtained from Wikipedia).

Edible and medicinal mushrooms are capable of stabilizing blood glucose level so as to avoid fatigue. Their hypoglycemic effect is achieved mainly through the increase of circulating insulin or relevant enzyme activities. For instance, Li and coworkers found that, with the administration of CSP-1, a kind of polysaccharides in* C. sinensis*, significant effect on blood insulin increase was observed. With the dose of 400 mg/kg body weight of CSP-1, the blood glucose level was reduced from 6.09 to 4.72 mmol/l and the insulin level was increased from 10 to 15.3 mlU/l after 7-day treatment [65]. Kiho et al. found that the alkaline extracts of* C. sinensis* had hypoglycemic effect through increasing the enzyme activities of glucokinase, hexokinase, and glucose-6-phosphate dehydrogenase [66]. Kiho and coworkers found similar effect from* Tremella aurantia* which could also improve relevant enzyme activities to regulate blood glucose level [67]. Increasing blood insulin and enhancing the activities of these enzymes can effectively increase cell glucose metabolic rate and promote blood oxygen and nutrient transport so as to mitigate fatigue.  Figure 2 summarizes the physiological basis of fatigue and the antifatigue mechanisms of edible and medicinal mushrooms in a more explicit way, with the detailed antifatigue mechanisms summarized in Table 2.

Previous studies have demonstrated that* C. sinensis* and* G. lucidum* are two representative antifatigue mushrooms because they can work in various biological systems. Based on the above review, their antifatigue mechanisms are summarized as follows.* C. sinensis* can increase glycogen storage and limit the accumulation of lactic acid in blood. It also enhances the expression of muscle metabolic regulators as well as lactate and glucose transporters to promote energy utilization and retard acidosis in muscle.* C. sinensis* can also scavenge free radicals generated during oxidative metabolism and stimulate vasodilation. Besides, it is also capable of improving energy state of liver, stimulating body testosterone level, and regulating blood glucose level. The cytokines expression and activities of immune cells including macrophages, natural killer cells, and T helper cells are enhanced by* C. sinensis* as well. All these effects support that* C. sinensis* can strongly enhance body fatigue resistance.

*G. lucidum* can increase glycogen storage, improve BUN clearance rate, limit lactic acid accumulation, and significantly improve LDH activity. It can also inhibit lipid peroxidation and scavenge nitric oxide and superoxide anion. Besides,* G. lucidum* is capable of stimulating macrophage activities and the expression of cytokines, as well as balancing body testosterone level. Vasodilation could also be induced by* G. lucidum*, which reduces blood viscosity, inhibits platelet aggregation, and improves blood transport efficiency. These pharmacological effects of* G. lucidum* make it an effective antifatigue mushroom as well.

## 4. Polysaccharides as Active Components of Edible and Medicinal Mushrooms for Antifatigue Effect

Polysaccharides are very important components in edible and medicinal mushrooms. The bioactive polysaccharides isolated from mushrooms include homopolysaccharides commonly extracted as glucans and heteropolysaccharides with different compositions and types of glycosidic linkages. Some are bound with protein or peptide residues forming polysaccharide-protein or polysaccharide-peptide complexes [68]. Polysaccharides in edible and medicinal mushrooms could mitigate fatigue through the improvement on muscular function, antioxidant effect, blood glucose regulation, immunomodulation, and hormone regulation.

### 4.1. Homopolysaccharides

**Figure 3:**
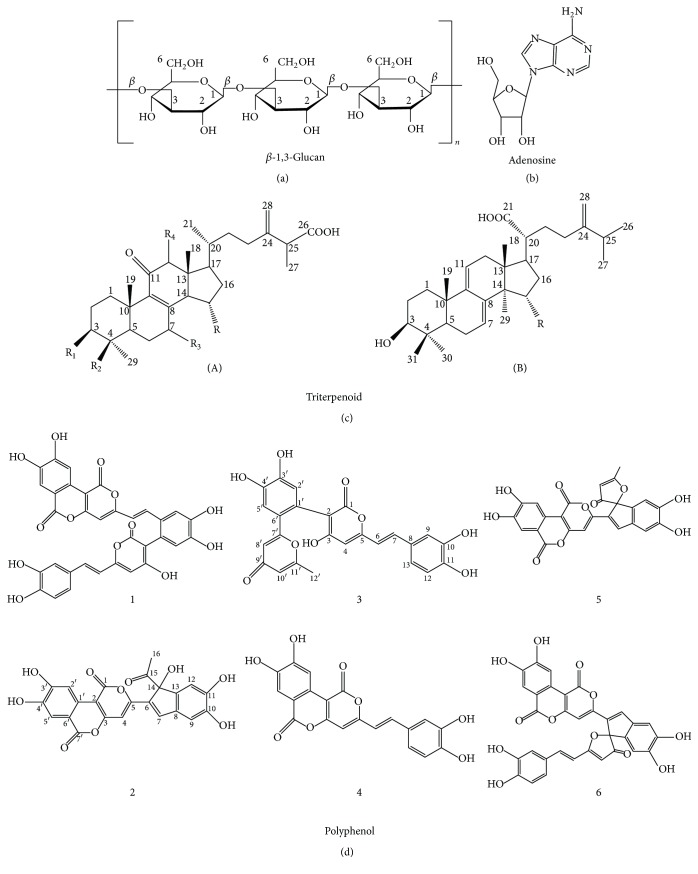
Typical structures of bioactive molecules in edible and medicinal mushrooms. (a) *β*-1,3-Glucan, (b) adenosine, (c) triterpenoids from* Antrodia camphorate* with (A) ergostane type and (B) lanostane type [12], and (d) polyphenol from* Inonotus obliquus* with 1, 2, 3 as inonoblins and 4, 5, 6 as phelligridins [44].

Homopolysaccharides have been isolated from edible and medicinal mushrooms by many researchers. The polysaccharide of* G. lucidum* was found to be *β*-D-glucan, which was a potent stimulator for macrophages, helping them with nitric oxide release to kill pathogens more effectively [55]. Zhonghui et al. found strong evidence that the polysaccharide from* G. lucidum* possessed protective effects against exhaustive exercise-induced oxidative stress through increasing antioxidant enzymes activities and decreasing malondialdehyde (MDA) levels in the skeletal muscles of mice [69]. The polysaccharides from* A. bisporus*, especially *β*-1,3-glucans (Figure 3(a)), were discovered as the bioactive molecules for immunomodulation enhancement, which functioned by improving the natural killer cell activities [59]. Bobovčák et al. found that the *β*-glucan from* Pleurotus ostreatus* could modulate exercise-induced changes in natural killer cell activities (NKCA) in intensively training athletes. They found that, after intense exercise, the placebo group showed a 28% reduction in NKCA below the baseline value, whereas the group treated with *β*-glucan from* P. ostreatus* had no significant reduction in NKCA [70]. A new water soluble glucan with a molecular weight of around 1.48 × 10^5^ Da was obtained by Maity and coworkers from the hot water extracts of* Meripilus giganteus*, showing hydroxyl and superoxide radicals scavenging activity [71]. Maity et al. isolated a water soluble *β*-glucan from the hot water extracts of* Entoloma lividoalbum* fruit bodies, which exhibited strong immunomodulation effect by stimulating macrophage, splenocyte, and thymocyte. Meanwhile, promising antioxidant activities of the isolated *β*-glucan were observed as evidenced from its hydroxyl and superoxide radicals scavenging activities [72].

### 4.2. Heteropolysaccharides

The polysaccharides isolated from edible and medicinal mushrooms could also be heteropolysaccharides. Polysaccharides from* C. sinensis* CSP-1 composed of glucose, mannose, and galactose in the ratio of 1 : 0.6 : 0.75 with molecular weight of 210 kDa demonstrated hypoglycemic effect by increasing glucose metabolic rate [65]. Other research showed that the polysaccharides from* C. sinensis* were effective in improving swimming endurance time of mice through inhibiting lactic acid production during exercise and increasing liver and muscle glycogen storage [32]. The body testosterone level could also be stimulated by polysaccharides of* C. sinensis* to boost body energy level [61]. A heteropolysaccharide of* T. fuciformis* with the backbone chain of *α*-(1→3)-linked D-mannan and small xylose- and glucuronic acid-containing side chains was found to stimulate the formation and activity of SOD and GSH-Px enzymes and effectively scavenge free radicals [48, 50]. The studies of Xin et al. found that* T. fuciformis* polysaccharides can help to prolong the time for skeletal muscles to become fatigued with the concentration of 0.10% treated muscle showing the longest time to be fatigue [73]. Meanwhile, the polysaccharides from* T. fuciformis* were also found to enhance transcription in liver cells, which promoted liver protein synthesis and helped to maintain the normal function of liver [64].* L. edodes* polysaccharides mainly consisting of mannose, glucose, and galactose were found to scavenge excess superoxide anion and hydroxyl radicals produced during aerobic respiration [74] and promote the production of antioxidant enzymes, especially GSH-Px [49]. Siu et al. purified two polysaccharides from wild* Armillaria ostoyae*. One was composed solely of glucose; the other was a branched galactoglucan with glucose and galactose at the molar ratio of 6 : 1, exhibiting a more significant antioxidant capacity [75]. Three types of polysaccharides were purified from* Flammulina velutipes* with molecular weights of 9,930, 62,290, and 36,310 Da, respectively, with the latter two showing higher antioxidant activities. All of these polysaccharides were heteropolysaccharides comprising glucose, galactose, mannose, and xylose with different molar ratios [76]. Manna and coworkers isolated a water soluble heteroglycan (Mw around 60 kDa) from the hot aqueous extract of* Lentinus fusipes*. The heteroglycan was found to be composed of D-galactose and D-glucose in the molar ratio of approximately 1 : 1, showing both* in vitro* splenocyte and macrophage activation and DPPH radical scavenging activity [77].

### 4.3. Polysaccharide-Protein or Polysaccharide-Peptide Complexes

Polysaccharide-protein or polysaccharide-peptide complexes isolated from edible and medicinal mushrooms also exhibit antifatigue functions. Zhang and coworkers investigated the polysaccharide extracts from* Ganoderma atrum* and figured out that its antioxidant activities were ascribed to the phenolic and protein components of the polysaccharide conjugates, while the immune-modulatory activity should be more attributed to the (1→3, 1→6)-linked-heteroglucan structure [78]. It was mainly composed of mannose, galactose, and glucose in the molar ratio of 1 : 1.28 : 4.91, with an average molecular weight of about 1,013 kDa [79]. Li and coworkers purified a novel 16 kDa polysaccharide-peptide complex from* Pleurotus abalones* with the monosaccharide composition characterized as glucose, rhamnose, glucuronic acid, and galactose in the molar ratio of 22.4 : 1 : 1.7 : 1.6. The peptide component with N-terminal amino acid sequence showed similarity to antioxidant enzymes, indicating that the antioxidant function of* P. abalones* may come from the peptide component of the polysaccharide-peptide complex [80]. Wu and coworkers studied* C. sinensis* Cs-HK1 and found that the carbohydrate moiety of the exopolysaccharide (EPS) was mainly composed of glucose and mannose at the mole ratio of 3.2 : 1.0. The glycopeptides component of the EPS with an average molecular weight of 6.0 kDa showed remarkable antioxidant capacity [81]. A summary of representative research on polysaccharides in edible and medicinal mushrooms for fatigue mitigation is shown in Table 3.

From the major functions of polysaccharides in edible and medicinal mushrooms such as inhibiting lactic acid formation, increasing glycogen storage, hormone regulation, blood glucose regulation, antioxidant, and immunomodulation, the body will function in a balanced way with efficient energy to fight against fatigue.

## 5. Other Bioactive Constituents of Mushrooms for Antifatigue Effect

### 5.1. Peptide and Proteins

The major roles of peptide and proteins of edible and medicinal mushrooms include the improvement on cardiovascular function, immunomodulation, and antioxidant effect. The polypeptide macromolecule in the aqueous phosphate buffer saline extracts of* C. sinensis* was found to stimulate nitric oxide production and EDHF to induce vasodilation effect and improve cardiovascular function. It redistributed blood to supply adequate amount of oxygen and nutrients to muscles and promoted blood circulation to remove waste products in muscles. The polypeptides also improved the energy state of liver, helping it with blood circulation and blood cleaning to carry more oxygen and nutrients throughout the body [53]. The protein, Ling Zhi-8 (LZ-8), found in* G. lucidum* extracts was found to help T lymphocytes to respond to cytokines more effectively so as to enhance the immunomodulation effect [84]. The peptides and crude proteins isolated from* G. lucidum* were also demonstrated to have antioxidant effect [11, 91]. Girjal and coworkers found that the amino acid composition of the peptide in* G. lucidum* was rich in phenylalanine, aspartic acid, proline, histidine, and isoleucine [11]. Geng and coworkers isolated an angiotensin converting enzyme inhibitory peptide from the crude water extracts of the fruiting bodies of* Tricholoma matsutake*, having antihypertensive effect to improve the cardiovascular function. This peptide was also demonstrated to have antioxidant effect as well through exhibiting DPPH radical scavenging activity [89].

### 5.2. Adenosine

Adenosine is a purine nucleoside consisting of ribose and adenine (Figure 3(b)). It is one of nucleotides of DNA and the molecular component of ATP, ADP, and AMP which have important roles in the biochemical processes like energy transfer and signal transduction. The studies on adenosine reported that it can reduce blood viscosity and improve blood oxygen supply capacity [54]. Adenosine has been found in many medicinal mushrooms such as* C. sinensis* and* G. lucidum*. Because adenosine hinders calcium ions uptake by muscle cells and causes intracellular interference, the contraction of smooth vessel muscles is inhibited, which triggers vasodilation and increment of oxygenated blood to the muscle cells [90]. Yoshioka and coworkers found that the adenosine isolated from* Grifola gargal* could promote glucose uptake in skeletal muscle cells [13]. The studies of Kawagishi et al. on* G. lucidum* adenosine suggested that adenosine can also have an inhibitory activity to platelet aggregation, which is the clumping of platelet in blood leading to the formation of clot [86]. The aggregation inhibition property of adenosine may help to enhance blood circulation and mitigate fatigue.

### 5.3. Triterpenoids

The studies of Zhu et al. suggested that the triterpenoids extracted from* G. lucidum* were the active components contributing to the antioxidant effect. The triterpenoids can significantly inhibit lipid peroxidation and prevent the formation of peroxynitrite through scavenging nitric oxide and superoxide anion [85]. They could also help to regulate the body testosterone level especially when the level was abnormally high by inhibiting the production of 5*α*-reductase which was an important enzyme to activate testosterone [62]. Huang and coworkers found that* Antrodia camphorate* also had a great proportion of triterpenoid compound, namely, ergostane and lanostane skeleton triterpenoids (Figure 3(c)), in the bioactive extracts. They had obvious antifatigue activity with dose-dependently increased swimming time, blood glucose, and muscular and hepatic glycogen levels as well as dose-dependently decreased plasma lactate, ammonia levels, and creatine kinase activity [12].

### 5.4. Phenolic/Flavonoid Compounds

Islam and coworkers studied 43 commonly consumed mushrooms in China and figured out a high correlation between the antioxidant capacity and phenolic/flavonoid content. Among these edible mushrooms,* Boletus aereus*,* Phellinus igniarius*,* Umbilicaria esculenta*,* Grifola frondosa*, and* Chroogomphus rutilus* have relatively higher total phenolic and flavonoid contents and show comparatively higher antioxidant capacities [14]. Researchers found that the methanolic extracts of* A. bisporus*, which contained gallic acid, flavonoid, ascorbic acid, and phenolic compounds, can act as free radical scavengers and contribute to its potent antioxidant activity [87, 88]. The polyphenol components isolated from the fruiting body of* Inonotus obliquus*, namely, inonoblins and phelligridins (Figure 3(d)), were also demonstrated to exhibit significant DPPH radical scavenging and moderate superoxide radical anion scavenging activities [44].

Besides the above-mentioned components, the mannitol and cordycepin in* C. sinensis* extracts were also found active for the scavenging of free radicals. Mannitol is a kind of sugar alcohol that commonly exists in bacteria, yeast, and mushrooms. It acted as a ROS quencher scavenging hydroxyl radicals generated by the pathogen defense system [82]. Cordycepin is a 3-deoxyadenosine which is the derivative of nucleoside adenosine with absence of oxygen in the 3′ position of its ribose. It also showed scavenging ability of free radicals like hydroxyl radicals and superoxide anion [83].

All the effects of those bioactive components from edible and medicinal mushrooms will help to regulate body balance and ensure a well-functioned body system to reduce the occurrence of fatigue.  Table 4 summarizes the active components of typical edible and medicinal mushrooms in Section 5 and their corresponding functions in fatigue mitigation.  Figure 3 further lists typical structures of active biological components in edible and medicinal mushrooms, including *β*-1,3-glucan, adenosine, triterpenoid, and polyphenol.

## 6. Concluding Remarks and Future Perspectives

There are four major actual sites of action for the antifatigue activities of edible and medicinal mushrooms, namely, the muscular system, body antioxidant system, cardiovascular system, hormone system, and immune system. For the muscular function, the mushrooms mainly affect liver and muscle glycogen storage and blood lactic acid, which are the major causes for muscular fatigue. For the antioxidant effect, the mushrooms enhance the body antioxidant system by stimulating enzymes such as SOD and GSH-Px as well as limiting the detrimental effect of ROS. For the immune and hormone systems, the antifatigue function is achieved by stimulating the activities of immune cells and the expression of cytokines, as well as the testosterone. Besides these major antifatigue effects, some specific medicinal effects of edible and medicinal mushrooms also help to avoid fatigue, through improvement of liver energy state and function, blood circulation, and blood glucose regulation. Overall these demonstrate that edible and medicinal mushrooms can promote the proper function and balance of the biological systems to maintain the basic harmonious pattern of body for antifatigue.

Among the various bioactive components found in edible and medicinal mushrooms, polysaccharides are the most common active components responsible for their activities, with their activities differing based on size, structure, and composition. In addition, some other specific bioactive components, such as peptides, adenosines, triterpenoids, polyphenols, and flavonoids, can also improve body functions through action on the antioxidant, cardiovascular, hormone, and immune systems.

Because most experiments have been carried out in animal models, the results are not all convincing to prove that edible and medicinal mushrooms can effectively deal with human fatigue. Meanwhile, other than polysaccharides, there are few results about the direct correlation of one specific bioactive component with the antifatigue effect, especially from the evaluation of muscular function improvement. Further studies should be performed on these aspects for better understanding of the mechanism for the antifatigue function of edible and medicinal mushrooms.

## Figures and Tables

**Table 1:** Antifatigue effects of some common edible and medicinal mushrooms on mice [35].

Group	Dosage (g/kg feed)	BUN (mg/dl)	Lactic acid (mg/dl)	LDH (U/dl)	Swimming time (min)
Control	0.0	42.0 ± 8.72	65.2 ± 19.41	442 ± 55.0	221.0 ± 68.4
*L. edodes*	60.0	35.1 ± 6.04	47.5 ± 12.50	465 ± 45.3	295.1 ± 95.8
*H. erinaceus*	60.0	36.2 ± 4.69	46.0 ± 13.62	440 ± 53.1	290.5 ± 89.2
*T. fuciformis*	60.0	37.4 ± 4.35	48.5 ± 12.45	445 ± 68.0	294.2 ± 84.3
*G. lucidum*	30.0	31.5 ± 5.15	46.8 ± 11.20	518 ± 50.2	319.2 ± 61.6
*C. sinensis*	8.0	32.7 ± 4.82	43.7 ± 13.00	504 ± 51.6	308.5 ± 69.3

**Table 2:** Summary of the antifatigue functions and possible mechanisms of mushrooms.

Antifatigue functions of mushrooms	Detailed mechanisms
Muscular function improvement	(i) Inhibits production and accelerate clearance of lactic acid; (ii) Increases glycogen storage in liver and muscle; (iii) Reduces blood urea nitrogen from protein breakdown; (iv) Increases lactate dehydrogenase activity; (v) Promotes the expression of skeletal muscle key metabolic regulators of AMPK, PGC-1*α*, and PPAR-*δ*; (vi) Stimulates the expression of lactate monocarboxylate transporter and glucose transporter.

Antioxidant function	(i) Scavenges DPPH, hydroxyl radical, superoxide anion radical, and nitric oxide; (ii) Inhibits lipid peroxidation; (iii) Stimulates antioxidant enzymes of SOD, GSH-Px, catalase, and glutathione reductase.

Cardiovascular function improvement	(i) Vasodilation in areas that need blood supply increase.

Immunomodulation enhancement	(i) Activates macrophages to engulf foreign invaders; (ii) Increases T helper cells to control and regulate other immune cells; (iii) Increases T helper: T suppressor cells to enhance immune system activity; (iv) Increases natural kill cells activity to identify and remove foreign invaders; (v) Improves cytokines expression to regulate cellular communication and immune response in infective sites.

Hormone regulation improvement	(i) Balances testosterone level to improve muscle development.

Hepatic function improvement	(i) Increases energy state by increasing ATP to Pi ratio; (ii) Increases liver blood flow; (iii) Promotes liver protein and RNA synthesis.

Blood glucose regulation improvement	(i) Increases circulating insulin to reduce glucose back to normal; (ii) Increases relevant enzyme activities including glucokinase, hexokinase, and glucose-6-phosphate dehydrogenase to increase glucose metabolic rate.

**Table 3:** Polysaccharides from edible and medicinal mushrooms for fatigue mitigation.

Family	Species	Polysaccharide composition	Mechanism for anti-fatigue	Ref.
Ganodermataceae	*Ganoderma lucidum*	*β*-D-Glucan	Stimulates macrophages activity; Promotes antioxidant enzyme activity.	[55, 69]

Agaricaceae	*Agaricus bisporus*	*β*-1,3-Glucan	Improves natural killer cell activities.	[59]

Pleurotaceae	*Pleurotus ostreatus*	*β*-Glucan	Modulates changes in natural killer cell activities.	[70]

Meripilaceae	*Meripilus giganteus*	Glucan, Mw of 1.48 × 10^5^ Da	Strong free radical scavenging activity.	[71]

Entolomataceae	*Entoloma lividoalbum*	*β*-Glucan	Stimulates macrophage, splenocyte, and thymocyte; Strong free radical scavenging activity.	[72]

Cordycipitaceae	*Cordyceps sinensis*	CSP-1 with glucose, mannose, and galactose in the ratio of 1 : 0.6 : 0.75; EPS-glycopeptide complex with EPS comprising glucose and mannose in the ratio of 3.2 : 1.0 and glycopeptide having Mw of 6.0 kDa	Hypoglycemic effect; Inhibits lactic acid production and increase liver and muscle glycogen storage; Regulates body hormone.	[32, 61, 65, 81]

Tremellaceae	*Tremella fuciformis*	*α*-(1→3)-linked D-mannan as backbone chain and small xylose- and glucuronic acid-containing side chains	Improves muscular function; Promotes antioxidant enzyme activity and strong free radical scavenging activity; Promotes liver protein synthesis.	[48, 50, 64, 73]

Marasmiaceae	*Lentinus edodes*	Mannose, glucose, and galactose	Strong free radical scavenging activity; Promotes antioxidant enzyme activity.	[49, 74]

Physalacriaceae	*Armillaria ostoyae*	Glucan and branched galactoglucan with glucose and galactose at the molar ratio of 6 : 1	Antioxidant activity.	[75]

Physalacriaceae	*Flammulina velutipes*	Three heteropolysaccharides with glucose, galactose, mannose, and xylose at different molar ratios and Mw of 9,930, 62,290, and 36,310 Da	Antioxidant activity.	[76]

Polyporaceae	*Lentinus fusipes*	Heteroglycan with D-galactose and D-glucose in the molar ratio of approximately 1 : 1 and Mw of 60 kDa	Stimulates splenocyte and macrophage activity; DPPH radical scavenging activity.	[77]

Polyporaceae	*Ganoderma atrum*	Polysaccharide-phenolic/protein conjugates with polysaccharide comprising mannose, galactose, and glucose in the ratio of 1 : 1.28 : 4.91 and Mw of 1,013 kDa	Antioxidant activity; Improves immunomodulation.	[78, 79]

Pleurotaceae	*Pleurotus abalones*	Polysaccharide- peptide complex with polysaccharide comprising glucose, rhamnose, glucuronic acid, and galactose in the molar ratio of 22.4 : 1 : 1.7 : 1.6 and peptide having N-terminal amino acid sequence	Antioxidant activity.	[80]

**Table 4:** Other bioactive constituents (than polysaccharides) and possible antifatigue mechanisms of some important mushrooms.

Family	Species	Active components	Mechanism for antifatigue	Ref.
Cordycipitaceae	*Cordyceps sinensis*	Polypeptide	Improves cardiovascular function through vasodilation effect; Improves energy state of liver.	[53]
		Mannitol	Free radical scavenging activity.	[82]
		Cordycepin	Free radical scavenging activity.	[83]

Ganodermataceae	*Ganoderma lucidum*	Protein of LZ-8	Improves T lymphocytes response to cytokines;	[84]
		Peptide with amino acids rich in phenylalanine, aspartic acid, proline, histidine, and isoleucine	Antioxidant activity.	[11]
		Triterpenoids	Strong antioxidant activity; Regulates body testosterone level.	[62, 85]
		Adenosine	Inhibitory platelet aggregation to enhance blood circulation.	[86]

Agaricaceae	*Agaricus bisporus*	Gallic acid, flavonoid, ascorbic acid, and phenolic compounds	Antioxidant activity.	[87, 88]

Tricholomataceae	*Tricholoma matsutake*	Peptide	Improves cardiovascular function by antihypertensive action; Free radical scavenging activity.	[89]

Meripilaceae	*Grifola gargal*	Adenosine	Promotes glucose uptake in skeletal muscle cells.	[90]

Fomitopsidaceae	*Antrodia cinnamomea*	Ergostane and lanostane skeleton triterpenoids	Significantly improves muscular function.	[12]

Hymenochaetaceae	*Inonotus obliquus*	Polyphenol of inonoblins and phelligridins	Significant DPPH radical scavenging and moderate superoxide anion scavenging activities.	[44]
